# Using Genetically Engineered Animal Models in the Postgenomic Era to Understand Gene Function in Alcoholism

**DOI:** 10.35946/arcr.v34.3.03

**Published:** 2012

**Authors:** Matthew T. Reilly, R. Adron Harris, Antonio Noronha

**Affiliations:** **Matthew T. Reilly, Ph.D.,***is a program director, Division of Neuroscience and Behavior, National Institute on Alcohol Abuse and Alcoholism, Bethesda, Maryland.*; **R. Adron Harris, Ph.D.,***is M. June and J. Virgil Waggoner Chair in Molecular Biology and director of the Waggoner Center for Alcohol and Addiction Research, University of Texas at Austin, Austin, Texas.*; **Antonio Noronha, Ph.D.,***is director of the Division of Neuroscience and Behavior, National Institute on Alcohol Abuse and Alcoholism, Bethesda, Maryland.*

**Keywords:** Alcoholism, genetic, genetic basis of alcoholism, neuroscience, gene function, genetic engineering, genetic technology, gene knockout technology, gene sequencing, gene functions, phenotype, genome, epigenome, transcriptome, animal studies, knockout mice

## Abstract

Over the last 50 years, researchers have made substantial progress in identifying genetic variations that underlie the complex phenotype of alcoholism. Not much is known, however, about how this genetic variation translates into altered biological function. Genetic animal models recapitulating specific characteristics of the human condition have helped elucidate gene function and the genetic basis of disease. In particular, major advances have come from the ability to manipulate genes through a variety of genetic technologies that provide an unprecedented capacity to determine gene function in the living organism and in alcohol-related behaviors. Even newer genetic-engineering technologies have given researchers the ability to control when and where a specific gene or mutation is activated or deleted, allowing investigators to narrow the role of the gene’s function to circumscribed neural pathways and across development. These technologies are important for all areas of neuroscience, and several public and private initiatives are making a new generation of genetic-engineering tools available to the scientific community at large. Finally, high-throughput “next-generation sequencing” technologies are set to rapidly increase knowledge of the genome, epigenome, and transcriptome, which, combined with genetically engineered mouse mutants, will enhance insight into biological function. All of these resources will provide deeper insight into the genetic basis of alcoholism.

During the first decade of the new millennium, remarkable advances in technology allowed investigators in all areas of biological research to collect massive amounts of genetic data at an unprecedented rate. The genomics revolution, which began with the sequencing of the human genome, was the basis for efforts such as the 1000 Genomes Project (www.1000genomes.org) that strive to compile a comprehensive catalogue of genetic variation in humans. A catalogue of genetic variation across multiple species also was borne out of this effort. Indeed, sequencing of the genome of the canonical research mouse strain, called C57BL/6, followed by the sequencing of other inbred mouse strains, has opened major opportunities for a fundamental understanding of how an organism’s genetic makeup (i.e., genotype) is related to its observable characteristics (i.e., phenotype). Sophisticated tools for creating genetically engineered animal models of human diseases also have reached a point where community-centered efforts have begun to eclipse previous efforts of individual laboratories. These genomic advances, coupled with major progress in genetic-engineering technology, are set to significantly enhance understanding of the genetic basis of human disease, including the genetic basis of alcoholism.

An inherited predisposition for alcoholism has been suspected for hundreds of years because of the observation that alcoholism tends to run in families. However, this familial pattern is not direct proof of a genetic vulnerability, because it also could be explained by a shared environment. It was not until the 1970s that this notion was scientifically tested in a systematic fashion, when Goodwin and colleagues (1973) studied the drinking histories of 55 adopted-out sons of alcoholics and 78 adopted-out sons of nonalcoholics, all of whom were adopted within the first 6 weeks of life. It is worth noting that the sons of alcoholics in this study had no knowledge that their biological parents had alcoholism. The results of this analysis were striking: The biological sons of alcoholics who had been adopted by nonrelated foster families were four times as likely to become alcoholics compared with the sons of nonalcoholics. Similar lines of research in twin and family studies convincingly have demonstrated that genetic factors account for between 50 to 60 percent of the vulnerability to alcoholism. However, although this statistic provides compelling evidence for a genetic influence on alcoholism, it does not indicate the specific genes that increase or decrease risk of developing alcoholism.

The search for genes associated with a predisposition toward alcoholism began more than 25 years ago. One of the first concerted research efforts to map such genes, the Collaborative Studies on the Genetics of Alcoholism (COGA), was established in 1989. The COGA sample is derived from more than 100 nuclear families densely affected with alcoholism, for whom extensive genotypic as well as phenotypic information has been collected. To date, researchers have identified about 20 genes that contribute to the risk of alcoholism in this sample. Similar studies by investigators all over the world, in a range of different populations, have identified additional genetic variants that contribute to the vulnerability to alcoholism. These genes encode proteins involved in almost all of the major brain-signaling (i.e., neurotransmitter) systems, including the γ-aminobutyric acid (GABA), glutamate, serotonin, dopamine, and acetylcholine systems. Genes involved with alcohol metabolism, other signaling mechanisms (e.g., neuropeptide and neuroendocrine signaling), and cellular architecture also have been implicated (Edenberg and Foroud 2006; Kranzler and Edenberg 2010). Yet, although this work has identified some candidate genes, it is only the first step in gaining insight into the etiology of alcoholism. The next step is to understand how genetic variation alters brain function and to determine which genes are most important for alcoholism and, finally, its treatment.

Understanding how the genotype of an organism is causally related to its phenotype is a fundamental goal of contemporary genetics. Over the last two decades, researchers have developed many innovative approaches to address this issue. Because of practical and ethical limitations associated with research in humans, the major thrust of this work has come from mechanistic studies in animal models of human diseases, particularly the use of genetically engineered animals. Indeed, animal models already have played a crucial role in understanding the genetic basis of alcoholism. The key approach used in these analyses is to manipulate genes in a controlled fashion in animal models in order to elucidate the genes’ function(s) in alcohol-related phenotypes. This review highlights recent advances in determining gene function using animal models with relevance to alcohol research. The discussion focuses almost exclusively on mouse models, because numerous novel genetic tools have been accumulating that allow gene manipulation in these models. In addition, this review describes publicly and privately funded community efforts for large-scale genetic engineering and systematic phenotyping. Finally, a brief introduction to novel high-throughput genomic sequencing technologies (e.g., next-generation sequencing) is presented. These technologies have great potential for furthering understanding of how genotype is causally related to phenotype by providing the most comprehensive depiction of the genome, transcriptome, and epigenome ever attempted.

## Conventional Genetic Strategies for Analyzing Gene Function

A widely used method to determine the function of a gene suspected of contributing to a certain trait (e.g., alcohol consumption) is to eliminate the gene from the organism under investigation (usually mice) through a method called homologous recombination. The resultant animal is called a null mutant or knockout for that gene. The investigator then determines what effect the absence of the gene (and the protein that it encodes) has on the trait being studied. Another conventional strategy for gene modification that uses the opposite approach to knockouts is called transgenesis. With this technique, a foreign gene (i.e., transgene) is introduced into a recipient organism’s genome, resulting in a transgenic animal. The product of the transgene can be produced at higher-than-normal levels (i.e., overexpressed) or otherwise manipulated in the transgenic animal in order to study the gene’s function. These approaches have been used extensively in alcohol research, and Crabbe and colleagues (2006) have published a comprehensive literature review covering the first 10 years (1996 to 2006) of studies using genetically engineered mice in this field. In addition, the Integrative Neuroscience Initiative on Alcoholism (INIA) West Consortium maintains a database containing historic and recent studies using genetically engineered mice in alcohol research.

Since 2007, numerous studies have used knockout mice to determine the effects of specific genes on alcohol consumption, using a standard two-bottle choice procedure in which the animals can freely choose between a water bottle and an alcohol (i.e., ethanol)-containing bottle for drinking (see table 1). In these studies, the knockout animals showed increases and decreases in ethanol drinking, depending on the specific gene that had been deleted. For one of the genes studied—a gene encoding a molecule called the CB1 receptor—studies consistently found that the knockout mice showed reduced ethanol drinking (Hungund et al. 2003; Naassila et al. 2004; Poncelet et al. 2003; Thanos et al. 2005). The results of other studies measuring ethanol consumption in animals in which genes such as those encoding proteins called adiponectin receptor 2, agouti-related protein, neurokinin-1 receptor, PSD-95, and adenylyl cyclase type 5 had been knocked out have yet to be confirmed in independent studies. Nevertheless, these initial findings offer exciting new possibilities for expanding the knowledge of the functional roles of genes associated with alcohol-related traits. For example, in a recent study, a group of neuroimmune genes were examined for their effect on ethanol consumption using knockout mice (Blednov et al. 2011*a*) (table 1). Previous genomics data measuring gene expression had implicated these genes in the response to alcohol. The results of the knockout studies demonstrate that these genes have a role in regulating alcohol consumption, thereby providing functional evidence supporting the initial gene expression studies. Thus, knockout studies can play a critical role in confirming the findings of other genomic studies and uncovering hitherto unknown molecular targets of ethanol.

However, the conventional knockout approach is associated with inherent limitations (for a detailed review of these limitations and ways to circumvent some of them, see Gerlai 1996; Wolfer et al. 2002). Briefly, one of the main limitations of studying conventional knockouts is the issue of developmental compensation. Because the gene of interest is inactivated over the entire lifespan of the knockout animal, changes in gene expression (or another biological response) in another or similar system may occur to compensate for the deleted gene. This compensatory response may obscure the real effects of the knockout on the trait of interest, resulting in false-negative results. Alternatively, any observed effects may result from the compensatory response rather than the actual gene knockout, leading to a false-positive effect. Another issue is background strain effects—that is, the effect of the knockout may vary depending on the mouse strain in which the knockout animal was generated. Finally, passenger-gene effects may occur. This means that during the process of genetically engineering a knockout animal, unintended genetic material can be introduced into the organism along with the genetic material required to create the knockout. These so-called passenger genes also can result in false-negative and false-positive effects. The next section describes some of the strategies used to overcome these limitations.

## Understanding Gene Function Through Conditional Knockout, Knockin, and Viral-Mediated Approaches

To overcome the limitations of conventional knockout studies, researchers have devised elegant and creative alternatives. Some of these strategies broadly can be classified as conditional strategies. The term “conditional” refers to the experimenter’s ability to impose specific time and space constraints on when and where the knockout or mutant is generated in the organism. This is accomplished, for example, by engineering engineering genetic elements that can be activated (i.e., induced) at a specific time by exposing the animal to a certain chemical agent. Another strategy is to engineer the knockout so that the gene only is deleted when a certain enzyme is present in the cell. By using enzymes that are expressed only in certain cells or tissues, the effects of the gene knockout also only would be limited to those cells or tissues.

Other approaches are using viruses to modify gene expression only in certain tissues. For example, viruses can be used to deliver inhibitory genetic material (referred to as RNA interference [RNAi]) directly to the brain, thereby allowing investigators to selectively reduce or “knockdown” the expression of target genes in specific brain areas. Conversely, viral-mediated approaches can help to overexpress a gene in a specified region.

Finally, as an alternative to eliminating an entire gene from an organism, researchers can introduce mutations into the gene that only change one or several amino acids in the protein that is encoded from the gene and determine the effect of this slight modification on function. This strategy is known as the knockin approach. Recently developed techniques even allow investigators to precisely control the timing and location of the expression of the knockin gene in the organism, resulting in a conditional knockin approach (Skvorak et al. 2006). One example of such a knockin approach, which will be discussed in more detail below, is a mutation in one of the genes encoding a component of the receptor for the neurotransmitter GABA. This modified variant of the GABA_A_ receptor subunit no longer responds to alcohol but retains its function as a GABA receptor. Use of this gene variant has allowed investigators to define the role of specific GABA receptors in the behavioral actions of alcohol without completely deleting the receptors (Blednov et al. 2011*b*; Harris et al. 2011; Werner et al. 2006). In particular, this approach demonstrated that alcohol acts on the α2 subunit of the GABA_A_ receptor to produce its activating and aversive behavioral effects (Blednov et al. 2011*b*). This result is intriguing because the gene encoding this subunit previously has been identified as a strong candidate gene for alcohol dependence in humans (Enoch 2008).

Alcohol researchers are just beginning to systematically apply these newer genetic-engineering approaches to their work, and the following paragraphs will illustrate a few recent examples, along with examples from related fields. Studies using the conventional knockout strategy found that global knockout of the gene encoding a brain enzyme called protein kinase C epsilon (PKCε) reduced alcohol self-administration as well as signs of alcohol withdrawal (Hodge et al. 1999; Olive et al. 2000). However, the specific brain region that mediated this effect was unknown, and effects of developmental compensation could not be ruled out conclusively. To address these issues, Lesscher and colleagues (2009) applied a conditional knockout strategy using viral vectors containing RNAi that were delivered directly into a brain region called the amygdala, thereby preventing expression PKCε in that region. Genetic knockdown of PKCε with these viral vectors significantly reduced alcohol consumption (Lesscher et al. 2009), indicating that PKCε expression in the amygdala is important for ethanol consumption in mice. This strategy enabled the investigators to not only rule out the issues of developmental compensation but also to determine the brain region where the PKCε gene exerted its effect.

Using a slightly different conditional knockout strategy, Brigman and colleagues (2010) examined the role of a glutamate receptor subunit in synaptic plasticity and learning, two phenomena that are critically involved in alcoholism. To generate the conditional glutamate receptor knockout animal, the researchers engineered a gene encoding the glutamate receptor subunit NR2B that would be removed only in the presence of a specific enzyme called Cre recombinase. Expression of the Cre recombinase, in turn, was restricted to the cortex and hippocampus by using a genetic element (i.e., promoter) called the CaMKII promoter that only is active in these brain tissues. As a result, deletion of the *NR2B* gene would be limited to those brain regions in which the CaMKII promoter was active. The *NR2B* conditional knockout mice showed significant impairments in a form of synaptic plasticity called long-term depression, altered morphology of nerve cells (i.e., neurons), and deficits in a learning task (Brigman et al. 2010). Because the *NR2B* gene knockout was restricted to the hippocampus and cortex, these brain regions obviously were crucial to the function of the *NR2B* gene. In addition, these analyses also partially controlled for development-specific factors of the genetic knockout because the CaMKII-driven expression of Cre recombinase occurs late in postnatal development. Thus, this approach eliminates any confounding effects that could be associated with the absence of the *NR2B* gene during earlier developmental stages.

The knockin strategy to understand gene function also has shown promise. The brain’s GABA-mediated (i.e., GABAergic) signaling system has been implicated in alcohol’s actions on the brain and also in mediating a genetic predisposition toward alcoholism (Enoch 2008). A recent study (Blednov et al. 2011*b*) used the knockin strategy to genetically modify the α2 subunit of the GABA_A_ receptor at just two amino acids. Mice carrying this α2 mutant still responded to GABA but failed to show enhanced GABA activity (i.e., potentiation) in response to alcohol. Behaviorally, α2 mutant mice failed to show alcohol-induced conditioned taste aversion and motor stimulation as well as displayed altered alcohol intake and preference. Therefore, using this knockin strategy, the researchers were able to support the α2 subunit’s role in specific actions of alcohol (Blednov et al. 2011*b*). Additional studies in these mice ruled out major developmental compensation effects of the mutated subunit (Harris et al. 2011), further confirm-

## Community Resources for High-Throughput Genetic Engineering

As the above examples indicate, genetic-engineering techniques hold tremendous power for dissecting the role of specific genes in alcohol-related phenotypes. The generation of genetically modified animals, however, requires a lot of time and resources, which can prevent investigators from creating needed mutant animals. Several community-wide resources have been developed to help facilitate the use of genetically engineered animal models for studying human diseases (table 2). This section briefly describes some of these resources.

### The Knockout Mouse Project (KOMP)

Both publicly and privately funded resources are available that aim to facilitate the use of genetically engineered animals to model human disease and understand gene function. The sequencing of several mouse genomes, including that of the widely used C57BL/6 strain, motivated the development of a resource to elucidate gene function. In 2003, a conference at the Banbury Center at the Cold Spring Harbor Laboratory discussed mouse genomics and genetic engineering, leading to an agreement to begin construction of a collection of mouse knockout mutants for every gene in the mouse genome (Austin et al. 2004). The strategy was to first generate null and conditional-ready knockout mutants in embryonic stem (ES) cells, using both gene-targeting and gene-trapping methodology. Next, mice would be generated from these ES cells to characterize the effects of the mutants at multiple levels of analysis, including gene expression and behavioral analyses. Another workshop, convened in Bethesda, Maryland, by the National Institutes of Health (NIH) in 2005, endorsed the proposals from the Banbury conference. This meeting launched a trans-NIH initiative called the Knockout Mouse Project (KOMP) As of May 2012, the KOMP now contains approximately 8500 targeted null mutations in the C57BL/6 mouse strain (http://www.nih.gov/science/models/mouse/knockout/). Several other similar domestic and international efforts also are being coordinated with the KOMP. These include the European Conditional Mouse Mutagenesis Program (EuCOMM) and the North American Conditional Mouse Mutagenesis Program (NorCOMM). Approximately 9000 knockout targeted alleles are available from these consortia. All three of these community efforts are coordinated under the International Knockout Mouse Consortium (IKMC) (http://www.knockoutmouse.org/).

### Knockout Mouse Project Phase 2 (KOMP^2^)

As the community-wide efforts to generate an extensive collection of null and conditional-ready mouse mutants are nearing the completion of their first phase, they are gearing up to begin generating mice for phenotyping. For example, the KOMP already has generated over 400 null mutant mouse lines and, at the current production rates, is set to produce a total of over 800. Storage of ES cells and production of mice are coordinated at a central repository, which is essential for ensuring that investigators rapidly can obtain animals and reagents for their research (http://www.komp.org/). The logistics of the phenotyping phase of the KOMP (i.e., the KOMP phase 2 [KOMP^2^]) were formalized at a workshop convened by the NIH in October 2009. The main recommendation was to initiate a coordinated high-throughput phenotyping effort for knockout mice generated by the KOMP, EuCOMM, and NorCOMM. The first phase of phenotyping was proposed to be broad based and conducted by a centralized phenotyping center. In addition, the gene list from the IKMC would be prioritized with the aim of discovering new phenotypes rather than confirming already-known knockout phenotypes. It was envisioned that the broad-based phenotyping program would draw on examples from other phenotyping efforts across Europe, such as the EmpressSlim model at MRC Harwell. This primary screen would include such phenotypes as measures of body weight, locomotion, pain sensitivity (i.e., nociception), and various immunological measures. Of particular interest to the alcohol research community, neurobehavioral measures also would be included in this stage. The broad-based phenotyping then would be followed by more specialized phenotyping by individual laboratories, which also would allow the alcohol research community to benefit greatly from this resource. For example, an alcohol researcher interested in gene X could obtain broad-based phenotyping data from the central repository for the knockout mouse generated for gene X, which would provide basic information on many baseline measures. The investigator then could design experiments in his or her own laboratory to test the knockout mouse for gene X on more specific measures, such as alcohol intake, withdrawal severity, and alcohol-induced motor stimulation. It is clear that this resource will provide an efficient means for alcohol researchers to discover novel genes associated with alcohol dependence.

Tang and colleagues (2010) recently published the results of such a systematic phenotyping approach using a large-scale mouse knockout library to elucidate gene function. In a tour de force, the researchers generated a mouse knockout library of 472 proteins that are either secreted by the cells or span the cell membrane (i.e., transmembrane proteins). The investigators reasoned that the genes encoding these proteins would be ideal knockout candidates, because the proteins are easily accessible therapeutic targets and understanding their function would be beneficial for drug development. The researchers performed systematic, broad-based phenotyping that encompassed several therapeutic areas, including embryonic development, metabolism, the immune system, the nervous system, and the cardiovascular system. Almost 90 percent of the knockout mutants (i.e., 419 mutants) showed an observable phenotype across various organ systems studied. Specifically, approximately 30 percent (i.e., 150 mutants) exhibited an observable phenotype in just one organ system, whereas approximately 60 percent exhibited a phenotype in two or more systems. This latter finding highlights the importance of pleiotropy—that is, the fact that a single gene can have more than one function so that a single mutation can give rise to multiple phenotypes. However, it is important to note that the mutants generated in this study all were conventional knockouts, and the caveats discussed above apply. Thus, although the approach is impressive for its large-scale effort in systematic phenotyping, it is difficult to draw clear conclusions from it because of potential developmental compensation on the phenotypes displayed by any particular knockout in this study. Community-wide efforts are under way to address some of these limitations. For example, large-scale projects to examine tissue- and cell-type– specific knockout animals are discussed below. Nonetheless, the study by Tang and colleagues (2010) is a seminal account demonstrating the utility of community-wide efforts to understand gene function using large-scale mouse knockout technology and systematic phenotyping.

### International Gene Trap Consortium: An Alternative Approach

As mentioned above, the community-wide effort to generate knockout ES cells for every gene in the mouse includes both the more common gene-targeting approach and gene-trapping technology. The International Gene Trap Consortium (IGTC) (www.genetrap.org) uses gene-trap technology for high-throughput mutagenesis to produce null mutants in mouse ES cells (Nord et al. 2006). It uses small DNA pieces (i.e., vectors) that simultaneously disrupt the target gene at the point of insertion and report the level of expression of the disrupted gene. Thus, gene trapping can produce gene variants (i.e., alleles) that entirely lose their function as well as a variety of other experimental alleles if newer gene-trap vectors are used that allow for modification of expression after the insertion. The IGTC oversees a repository of all publicly available gene-trap cell lines, which are freely available to investigators. Initially, there was some skepticism regarding the percentage of gene traps that could produce true null mutants and the fraction of the genome that ultimately can be covered by gene-trap mutations. The first attempts using this approach estimated a mutational coverage of approximately 60 percent of the mouse genome (Skarnes et al. 2004; Zambrowicz et al. 2003). More recent attempts, however, have shown more than 90 percent mutational coverage of the mouse genome (Gragerov et al. 2007). Thus, both gene-targeting and gene-trapping approaches are proving to be efficient high-throughput means of generating a community-wide resource of genetically engineered mice for studies of human disease.

### Cre-Driver Mouse Project

Another gene-targeting system that has provided investigators with extraordinary control of experiments to determine gene function in living organisms is called the Cre/loxP system, which already was alluded to earlier in this article. It allows for inducible and conditional gene targeting in the mouse by engineering the bacterial gene that encodes Cre recombinase into a mouse. Expression of the Cre recombinase then can be spatially restricted by fusing the gene with a cell- or tissue-specific promoter. Cre recombinase recognizes and cuts a short bacterial DNA segment called a loxP site. These sites can be genetically engineered into a separate mouse line (referred to as a floxed line or “conditional ready” line), in which the loxP sequences are placed strategically around a critical genomic region containing a gene or gene segment of interest. When animals from the Cre-line are crossed with animals from the floxed line, the gene or gene segment of interest is excised in a specific cell type or tissue, depending on the promoter used to control the Cre gene, and researchers can study the resulting effects.

As mentioned earlier, this system has been used to elucidate the function of a glutamate receptor subunit by using a cell-type–specific promoter to drive Cre recombinase expression in a restricted brain area. In addition, the Cre/loxP system has been used in several community-wide efforts to analyze gene function. One such key community-wide project is the Cre-driver network established by the NIH Neuroscience Blueprint initiative (http://www.credrivermice.org/index). This project was motivated by a major bottleneck in the process of establishing Cre/loxP lines—that is, a lack of efficient animal lines in which the Cre gene is under the control of different promoters (i.e., Cre-driver lines) resulting in differential patterns of spatial expression, particularly in the brain. Establishing these lines is particularly challenging because of the marked differences in gene expression among various brain regions (Sandberg et al. 2000), and the Cre-driver network was spawned by the research community’s need for a resource of Cre-driver lines that can be used for spatial/temporal and/or inducible knockout studies. To this end, the NIH Blueprint for Neuroscience Research funded three centers in the United States to generate genetically modified C57BL/6 mice expressing Cre recombinase in the nervous system. The resources generated from these projects will be made freely available to the neuroscience community. To date, more than 200 novel Cre-driver lines have been constructed, and many investigators are expected to use this resource as more Cre-lines are produced. Similar Cre-driver-line projects that are funded by private sources also are becoming available to the research community. For example, the Allen Institute for Brain Science reported on a robust and high-throughput Cre-reporting and characterization system for the whole mouse brain (Madisen et al. 2010).

One of the challenges inherent in using the Cre/loxP system for conditional gene modification is to verify the actual pattern of deletion of the gene of interest. This problem partially can be resolved by genetically engineering the mice so that they also express an easily measurable reporter gene (e.g., β-galactosidase or a fluorescent probe such as green fluorescent protein [GFP]) that is activated after Cre-mediated excision of a transcriptional stop signal. The expression of the reporter gene then can be visualized in the brain as a measure of the deletion pattern of a Cre-expressing line. These Cre reporter lines also are useful for mapping neuronal circuitry, imaging, and tracking cell populations in the intact organism. However, although reporter lines are useful as a first approximation of the pattern of Cre-mediated recombination, not all “floxed” reporter genes and target genes yield the same result. For example, the pattern of Cre-mediated recombination sometimes is specific to the floxed gene, and cautious interpretation of reporter line results therefore is warranted. Nevertheless, the Allen Institute for Brain Science is continuing to produce and characterize novel Cre reporter lines and making this data public through an online database (http://transgenicmouse.alleninstitute.org). This resource undoubtedly will enable investigators to determine the usefulness of various Cre-driver lines for cell-type–specific genetic manipulations.

### The Gene Expression Nervous System Atlas

The Gene Expression Nervous System Atlas (GENSAT) is another remarkable project that aims to catalogue gene-expression patterns of the developing and adult central nervous system in the mouse (http://www.gensat.org/index.html). This is accomplished by using a fluorescent reporter (such as GFP) to replace the coding region of a gene of interest in a bacterial artificial chromosome (BAC) that also carries the regulatory regions (e.g., promoter) required for the gene’s expression in the brain. These BAC constructs are injected into mouse eggs, and transgenic animals are generated. Using fluorescence microscopy, investigators then can visualize where the reporter gene is expressed, which reflects the natural expression of the gene of interest. To date, over 500 genes have been analyzed using this approach. This atlas of brain gene expression has significant implications for understanding the great variety of neuronal cell types (Gong et al. 2003). In addition to being a community resource for cataloging cell-type–specific gene expression in the brain, GENSAT has targeted Cre recombinase to specific neuronal populations using the BAC approach (Gong et al. 2007), thereby allowing investigators to use the lines for genetic manipulations, such as producing inducible or conditional knockout mice.

## High-Throughput Novel Genomic Sequencing

Although substantial progress has been made in identifying some of the genes associated with the risk for alcoholism, much of the genetic variation that contributes to alcoholism has yet to be identified because of the heterogeneous nature of the disease. However, the advent of novel high-throughput genomic-sequencing technologies that have become available within the last decade likely will accelerate this progress. These new sequencing technologies are termed “next-generation sequencing,” to distinguish them from the conventional sequencing technologies developed in the 1970s. The greatest improvements in these new technologies are massive increases in speed and an exponential drop in cost to sequence. Thus, next-generation sequencing machines can read up to 250 billion DNA building blocks (i.e., bases) per week, compared with approximately 25,000 per week using conventional sequencing. In addition, the price per base for sequencing has dropped approximately 100,000-fold over the last decade, which makes next-generation sequencing a realistic application for all areas of biology, including sequencing of large cohorts of humans and other experimental organisms. This new approach also has the power to rapidly uncover variation in non–protein-coding regions of the genome (e.g., regulatory regions or microRNAs) and to characterize all isoforms of a particular gene by detecting alternatively spliced variants.^1^ Another application of this new technology will be the complete depiction of the transcriptome and epigenome, which will have important implications for understanding how the genome functions in normal and pathophysiological conditions such as alcoholism. Creating comprehensive whole-genome maps that contain detailed information on genomic, epigenomic, and transcriptomic variation associated with alcohol dependence would greatly advance the alcohol research field.

With the advances in sequencing technologies and the resulting acceleration of data generation, the rate-limiting step of fully realizing the potential of this information now has become data analysis and bioinformatics, and it is quite clear that novel analytical approaches must be developed for meaningful data interpretation. An obvious way to address this issue would be to use a systems-based approach to interpret genomic data, including novel methods to analyze and detect gene–gene interactions (i.e., epistasis). In addition, because complete genomic information (including noncoding regulatory regions) will be at hand, novel methods to understand gene regulation are essential.

Finally, another important variable in determining vulnerability to alcohol dependence is the environment. Environmental factors can contribute as much as one-half of the total risk for developing alcoholism. However, this has not been studied systematically in relationship to gene-by-environment interactions, at least in part because of an incomplete knowledge of the genome. Next-generation sequencing, with its massive output of genomic data, likely will change this scenario by providing a foundation on which the effects of environmental perturbations can be assessed on a grand scale.

## An Exciting Future for Alcohol Genetics

The postgenomic era has seen the development of global efforts to understand the function of the genome. Over the last 10 years, international research consortia have been created to tackle this enormous task, and this model is proving to be efficient for highthroughput science. In particular, concerted efforts to knock out every gene in the mouse genome are succeeding because of the use of focused resource centers. The alcohol research community is just beginning to use these resources and stands to benefit greatly from them. For example, with the availability of knockout lines for every gene, it will be possible to define the genes responsible for specific actions of alcohol. In addition, readily available conventional and conditional knockout animals will advance quantitative trait locus mapping studies. In particular, conditional knockout studies will become more abundant in the alcohol research community, allowing investigators to avoid some of the major interpretative difficulties associated with the conventional knockout studies that have dominated in the last 15 years.

Another exciting possibility is the use of new animal genetic-engineering techniques to reproduce specific genetic changes seen in human alcoholics. For example, as described above, detailed genetic sequencing of both DNA and RNA (i.e., the genome and transcriptome) from many humans now is feasible, owing to the rapidly decreasing cost of next-generation DNA sequencing. This will lead to the discovery of changes in gene sequence or gene expression that are candidates for differences in the development of alcohol dependence. These same genomic changes then can be introduced into mice or other animal models by knockin, transgenic, or other approaches (see figure). This approach already has shown promise in the alcohol field. For example, one of several variants (i.e., polymorphism) of the gene encoding the μ-opioid receptor is associated with enhanced subjective responses to alcohol in humans and differentially affects treatment response to naltrexone (Ray and Hutchinson 2007). To directly deter-subjective responses to alcohol in humans and differentially affects treatment response to naltrexone (Ray and Hutchinson 2007). To directly determine the functional consequences of this polymorphism, a knockin mouse was generated that harbors the human allele. The results indicate that the knockin mouse shows a greater brain dopamine response after alcohol challenge, possibly providing a mechanism by which the human variant of the μ-opioid receptor affects drinking (Ramchandani et al. 2010). Additional characterization of this “humanized” mouse model surely will provide important information about the functional consequences of this polymorphism on alcohol behaviors.

Besides generating mice with human-specific polymorphisms in known genes via the knockin approach, genetic engineering could be used to manipulate the vast array of noncoding regions of the genome that are copied into mRNA (i.e., are transcribed) but do not encode a specific protein. For example, recently discovered large noncoding RNAs (lncRNAs) are known to have a critical role in maintaining the state of the DNA–protein complex (i.e., chromatin) that makes up the chromosomes. Chromatin states influence gene expression on a fundamental level (Khalil et al. 2009). Although it has not been attempted yet, genetically manipulating these lncRNAs in an animal model could uncover significant functional roles in alcohol-related behaviors.

Finally, the human and mouse genomes are estimated to contain approximately 25,000 genes. However, the number of alternative forms of these known genes (i.e., alternatively spliced variants) may be about 10 times this amount, creating substantial genomic diversity with unknown function. Genetic manipulation of the roughly 200,000 alternatively spliced gene variants has not been explored systematically. This area also holds tremendous potential for discovering novel relationships between genotype and phenotype by generating genetically engineered animal models with alternatively spliced gene variants. Given understanding the genetic basis of alcoholism are on the horizon.

## Figures and Tables

**Figure:**
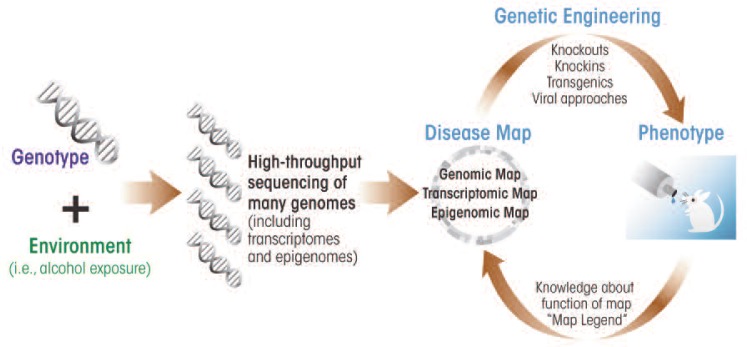
Exploring the relationship between genotype and phenotype by using high-throughput sequencing and genetically engineered animal models. Novel high-throughput “next-generation sequencing” technology can be used together with new genetic engineering technology to understand gene function in alcoholism. Compared with traditional sequencing, “next-generation sequencing” allows researchers to efficiently and cost-effectively obtain large amounts of genomic data (e.g., from large cohorts of humans with and without disease) to detect all the genomic, epigenomic, and transcriptomic variation associated with the disease, creating comprehensive “disease maps.” In a next step, functional information can be attached to these disease maps that defines how the various components of the map (i.e., individual genes) act and interact, for example, using genetically engineered animal models. Genomic variations associated with human diseases can be engineered into rodent models (or other experimental organisms) and detailed phenotypic analyses can be performed, further refining disease maps with functional annotation.

**Table 1:** Examples of Knockout Mouse Studies Conducted Between 2007 and 2011 That Demonstrate the Effects of the Deletion of Various Genes on Ethanol Consumption

**Knocked-Out Gene**	Result*	**Reference**
CB1 receptor	↓drinking	Vinod et al. 2008
δOpioid receptor	↑drinking	van Rijn and Whistler 2009
GABA A receptor α1	↓drinking	June et al. 2007
PKCε	↓drinking	Wallace et al. 2007
Adiponectin receptor 2	↓drinking	Repunte-Canonigo et al. 2010
Agouti-related protein	↓drinking	Navarro et al. 2009
Neurokinin-1 receptor	↓drinking	Thorsell et al. 2010
PSD-95	↓drinking	Camp et al. 2011
Adenylyl cyclase type 5	↑drinking	Kim et al. 2011
Beta-2-microglobulin	↓drinking	Blednov et al. 2011*a*
Cathepsin S	↓drinking	Blednov et al. 2011*a*
Cathepsin F	↓drinking	Blednov et al. 2011*a*
Interleukin 1 receptor antagonist	↓drinking	Blednov et al. 2011*a*
CD14 molecule	↓drinking	Blednov et al. 2011*a*
Interleukin 6	↓drinking	Blednov et al. 2011*a*

* Ethanol consumption was measured in a two-bottle choice (ethanol vs. water) paradigm

NOTE: For a complete catalogue of studies (1996–2006) on the use of genetically engineered mice in alcohol research see Crabbe et al. 2006.

**Table 2:** Community-Wide Resources for Genetically Engineered Mouse Models

**Resource**	**Description**	**Website**
Knockout Mouse Project (KOMP)	NIH initiative with the aim of generating mouse knock-outs in ES cells for every gene	www.nih.gov/science/models/mouse/knockout/
International Knockout Mouse Consortium (IKMC)	Consortium that coordinates international effort to produce mouse null mutants in ES cells for every gene	www.knockoutmouse.org
Knockout Mouse Project Phase 2 (KOMP^2^)^1^	Project aimed at producing knockout mice from ES cells and conducting broad-based phenotyping on them; knockout mice can be obtained at a central repository by individual investigators for use in their own laboratories	www.komp.org
International Gene Trap Consortium (IGTC)	Consortium that coordinates the international effort to use gene-trap technology for generating knockout mouse lines	www.genetrap.org
Cre-driver network	NIH initiative to produce Cre-driver mouse lines for conditional mouse knockout studies	www.credrivermice.org
Gene Expression Nervous System Atlas (GENSAT)	Project aimed at cataloguing gene expression patterns in the mouse central nervous system as well as providing a collection of Cre mouse lines.	www.gensat.org

1 The KOMP phase 2 (KOMP^2^) has just been initiated and resources generated by this project will be available in the near future.

## Footnotes

1 During gene expression, a “copy” of the gene, called the messenger RNA (mRNA) first is generated, which then is modified (i.e., spliced) to eliminate all sequences that do not encode the final protein product. For many genes, however, more than one splicing pattern exists, resulting in several variants, or isoforms, of the gene.
